# Supplementary material for: Circulating hs-CRP, IL-18, Chemerin, Leptin, and Adiponectin Levels Reflect Cardiometabolic Dysfunction in Adults with Excess Weight
Source: Int J Mol Sci. 2025 Jan 29;26(3):1176. doi: 10.3390/ijms26031176 (PMC11818792; doi:10.3390/ijms26031176)
Supplement: Supplementary file 1 [file ijms-26-01176-s001.zip › ijms-3403606-supplementary.pdf]

Table S1. Health information, anthropometry, blood chemistry, and gut microbiota of subjects categorized according to their waist circumference. The data are presented as the mean ± standard deviation (SD) and 95% confidence intervals (CIs). To compare groups, a multiple linear regression model (MLR) with log-transformed variables was used. The model was adjusted for age range, sex at birth, smoking and city of origin. Reported p-values <0.05 indicate the statistical significance of these comparisons.

	**Adequate waist Mean ± SD**	**CI 95%**	**Abdominal obesity Mean ± SD**	**CI 95%**	**p-value**	
N	41		75		Model 1	Model 2
Demographics						
Age (Years)	37.24 ± 11.27	33.69; 40.8	42.47 ± 11.48	42.47; 11.48	-	-
Female						
Male						
Anthropometry						
BMI (kg/m^2)	23.41 ± 2.20	22.72; 24.11	**30.19 ± 3.90**	29.29;31.08	**<0.0001**	**<0.0001**
Weight (kg)	60.40 ± 7.60	58.00; 62.80	**82.69 ± 12.81**	79.74; 85.64	**<0.0001**	**<0.0001**
Waist circumference (cm)	79.52 ± 4.61	78.07; 80.97	**101.44 ± 9.03**	99.36; 103.52	**<0.0001**	**<0.0001**
Body Fat (%)	34.40 ± 4.79	32.89; 35.91	**38.87 ± 4.92**	37.74; 40.00	**<0.0001**	**<0.0001**
Blood pressure						
Systolic (mm Hg)	116.98 ± 17.18	111.55; 122.40	**130.28 ± 18.77**	125.96; 134.60	**<0.0001**	**0.007**
Diastolic (mm Hg)	72.95 ± 12.03	69.16; 76.75	**83.25 ± 11.40**	85.88; 83.25	**<0.0001**	**<0.0001**
Blood lipids						
HDL (mg/dL)	49.56 ± 9.26	46.64; 52.48	**41.65 ± 10.94**	39.14; 44.17	**<0.0001**	**<0.0001**
VLDL (mg/dL)	22.28 ± 9.66	19.24; 25.33	**33.85 ± 25.36**	28.08; 39.69	**0.001**	**0.026**
LDL (mg/dL)	124.15 ± 36.84	112.52; 135.77	113.57 ± 30.70	106.45; 120.68	0.144	0.110
Total cholesterol (mg/dL)	193.68 ± 41.64	180.54; 206.83	186.09 ± 37.51	177.46; 194.72	0.343	0.142
Triglycerides (mg/dL)	111.51 ± 48.43	92.23; 126.80	**170.27 ± 126.49**	141.16; 199.37	**<0.0001**	**0.018**
ox-LDL (U/L)	153.29 ± 69.48	131.07; 175.51	176.82 ± 119.30	148.59; 205.06	0.484	0.667
ApoB (mg/dL)	92.09 ± 23.75	84.60; 99.59	104.40 ± 74.74	87.21; 121.60	0.309	0.623
Blood sugar control						
HbA1c (%)	5.38 ± 0.29	5.29; 5.47	**5.55 ± 0.47**	5.44; 5.65	**0.050**	**0.039**
Glucose (mg/L)	84.20 ± 7.97	81.68; 86.71	**90.87 ± 15.49**	87.30; 94.43	0.060	**0.050**
Insulin (µU/ml)	9.64 ± 4.61	8.19; 11.10	**15.63 ± 9.75**	13.38; 17.87	**<0.0001**	**<0.0001**
HOMA-B	184.17 ± 113.14	148.46; 219.88	**234.19 ± 163.28**	196.63; 271.76	0.123	**0.003**
HOMA-S	61.39 ± 27.67	52.66; 70.13	**40.26 ± 23.33**	34.89; 45.63	**<0.0001**	**<0.0001**
HOMA-IR	2.01 ± 1.00	1.70; 2.33	**3.57 ± 2.49**	3.00; 4.15	**<0.0001**	**<0.0001**
Gut microbiota and metabolites						
CAG-Prevotella	0.14 ± 0.20	0.08; 0.20	0.18 ± 0.24	0.12; 0.23	0.530	0.783
CAG-Lachnospiraceae	0.19 ± 0.20	0.13; 0.26	0.25 ± 0.24	0.20; 0.31	0.385	0.809
CAG-Pathogen	0.10 ± 0.19	0.04; 0.16	**0.22 ± 0.28**	0.16; 0.28	**0.018**	**0.047**
CAG-Akkermansia	0.29 ± 0.29	0.19; 0.38	**0.13 ± 0.19**	0.08; 0.17	**<0.0001**	**0.010**
CAG-Ruminococcaceae	0.13 ± 0.15	0.09; 0.18	**0.09 ± 0.13**	0.06; 0.12	**0.009**	**0.018**
TMA (µM)	1.91 ± 0.62	1.72; 2.11	1.84 ± 0.67	1.68; 1.99	0.423	0.354
TMA-O (µM)	3.67 ± 0.55	3.50; 3.85	3.65 ± 0.55	3.53; 3.78	0.854	0.740
Adipokines and inflammation markers						
hsCRP (mg/L)	1.62 ± 1.09	1.27; 1.96	**3.83 ± 5.48**	2.57; 5.09	**<0.0001**	**<0.0001**
TNF-α (pg/ml)	11.86 ± 5.31	10.18; 13.53	**14.98 ± 8.99**	12.91; 17.05	**0.034**	**0.037**
IL-6 (pg/ml)	4.97 ± 3.68	3.81; 6.13	**6.66 ± 5.71**	5.34; 7.97	0.059	**0.040**
IL-33 (pg/ml)	124.33 ± 17.12	118.92; 129.73	130.43 ± 19.09	126.04; 134.82	0.093	0.210
IL-8 (pg/ml)	28.74 ± 39.33	16.32; 41.15	42.75 ± 77.17	24.99; 60.50	0.209	0.073
MCP-1 (pg/ml)	235.18 ± 115.46	198.74; 271.63	284.7 ± 131.2	254.52; 314.89	**0.020**	0.066
IL-1β (pg/ml)	11.25 ± 1.87	10.66; 11.84	11.79 ± 2.45	11.22; 12.35	0.320	0.129
Visfatin (ng/ml)	2.70 ± 2.75	1.84; 3.57	2.95 ± 2.65	2.34; 3.56	0.347	0.211
Resistin (ng/ml)	12.78 ± 4.71	11.30; 14.27	12.93 ± 5.86	11.58; 14.28	0.778	0.735
Lipocalin-2 (ng/ml)	28.86 ± 4.20	27.54; 30.19	28.63 ± 5.17	27.44; 29.82	0.645	0.400
CXCL5 (ng/ml)	1.37 ± 0.88	1.09; 1.65	1.44 ± 1.09	1.18; 1.69	0.869	0.515
Chemerin (ng/ml)	7.60 ± 2.10	6.94; 8.26	**9.32 ± 3.68**	8.47; 10.17	**0.020**	**0.049**
Vaspin (ng/ml)	2.30 ± 6.75	0.17; 4.43	3.54 ± 9.12	1.44; 5.64	0.460	0.810
IL-18 (pg/ml)	239.30 ± 96.93	208.71; 269.9	**315.01 ± 121.90**	286.96; 343.06	**<0.0001**	**0.019**
Leptin (ng/ml)	5.47 ± 5.11	3.85; 7.08	**8.04 ± 7.13**	6.40; 9.68	**0.037**	**<0.0001**
Adiponectin (µg/ml)	8.06 ± 3.80	6.86; 9.26	**5.35 ± 2.66**	4.73; 5.96	**<0.0001**	**<0.0001**
Adiponectin/Leptin	4.08 ± 5.02	2.49; 5.66	**1.62 ± 2.12**	1.13; 2.11	**<0.0001**	**<0.0001**

BMI: body mass index; HDL: high-density lipoprotein cholesterol; VLDL: very low-density lipoprotein cholesterol; LDL: low-density lipoprotein cholesterol; oxLDL: oxidized low-density lipoprotein cholesterol; ApoB: Apolipoprotein B; TMA: Trimethylamine; TMA-O: Trimethylamine N-oxide; HbA1c: glycated hemoglobin; HOMA: homeostasis model assessment; CAG co-abundance groups; hsCRP high sensitivity C reactive protein; TNF-α: Tumor Necrosis Factor alpha; IL: Interleukin; MCP-1: Monocyte Chemoattractant Protein-1; CXCL5: C-X-C motif chemokine ligand 5. Model 1. MLR unadjusted. Model 2 MLR adjusted for potential confounders age range, sex at birth, smoking and city of origin.

Table S2. Health information, anthropometry, blood chemistry, and gut microbiota of subjects categorized according to their body mass index. The data are presented as the mean ± standard deviation (SD) and 95% confidence intervals (CIs). To compare groups, a multiple linear regression model (MLR) with log-transformed variables was used. The model was adjusted for age range, sex at birth, and city of origin was used. Reported p-values indicate the statistical significance of these comparisons. Post hoc analyses employing Dunn's test were conducted for individual contrasts. Differences were considered statistically significant if the adjusted p-value was less than 0.05. (*) indicates a statistically significant difference between individuals with adequate weight and those with overweight or obesity, (δ) denotes a significant difference between individuals with overweight and obesity.

	**Adequate weight**		**Overweight**		**Obesity**		**p - value**	
	**Mean ± SD**	**CI 95%**	**Mean**	**CI 95%**	**Mean**	**CI 95%**		
**N**	35		49		32		Model 1	Model 2
Demographics								
Age (years)	39.5		40.2		42.5			
Female	18		28		17			
Male	17		21		15			
Anthropometry								
BMI (kg/m^2)	22.63 ± 1.53	22.11; 23.16	**27.60 ± 1.28***	27.23; 27.97	**33.72 ± 3.21***$^{\delta}$	32.57; 34.88	**<0.0001**	**<0.0001**
Weight (kg)	60.46 ± 8.82	57.43; 63.49	**74.39 ± 8.88***	71.84; 76.94	**91.15 ± 13.24***$^{\delta}$	86.37; 95.92	**<0.0001**	**<0.0001**
Waist Circumference (cm)	80.21 ± 6.76	77.88; 82.53	**93.53 ± 6.79***	91.58; 95.48	**108.68 ± 8.61***$^{\delta}$	105.58; 111.79	**<0.0001**	**<0.0001**
Body Fat (%)	33.76 ± 4.67	32.15; 35.36	**36.93 ± 4.31***	35.69; 38.17	**41.71 ± 4.15***$^{\delta}$	40.22; 43.20	**<0.0001**	**<0.0001**
Blood pressure								
Systolic BP (mm Hg)	115.89 ± 12.1	111.73; 120.04	**129.88 ± 22.33***	123.46; 136.29	**129.59 ± 16.96***	123.48; 135.71	**0.001**	**<0.0001**
Diastolic BP (mm Hg)	72.89 ± 9.26	69.71; 76.07	**82.65 ± 14.13***	78.59; 86.71	**82.31 ± 10.55***	78.51; 86.12	**<0.0001**	**<0.0001**
Blood lipids								
HDL (mg/dL)	50.86 ± 10.67	47.19; 54.52	**42.20 ± 8.96***	39.63; 44.78	**40.88 ± 11.53***	36.72; 45.03	**<0.0001**	**<0.0001**
VLDL (mg/dL)	21.98 ± 9.30	18.78; 25.17	**30.17 ± 18.66***	24.81; 35.52	**37.67 ± 31.66***	26.25; 49.08	**0.003**	**<0.0001**
LDL (mg/dL)	126.06 ± 38.02	113.00; 139.12	113.73 ± 30.92	104.85; 122.62	114.88 ± 31.09	103.66; 126.09	0.264	0.357
Total cholesterol (mg/dL)	196.31 ± 42.83	181.60; 211.03	183.86 ± 36.00	173.52; 194.2	188.06 ± 39.04	173.99; 202.14	0.395	0.398
Triglycerides (mg/dL)	109.89 ± 46.53	93.90; 125.87	**152.59 ± 93.01***	125.88; 179.31	**188.09 ± 158.37***	131.00; 245.19	**0.003**	**<0.0001**
ox-LDL (U/L)	154.67 ± 68.34	131.19; 178.14	161.79 ± 79.01	139.10; 184.48	196.71± 153.74	141.28; 252.14	0.629	0.522
ApoB (mg/dL)	96.42 ± 27.25	87.06; 105.78	105.83± 90.64	79.80; 131.87	95.17± 23.79	86.60; 103.75	0.974	0.989
Blood sugar control								
HbA1c (%)	5.39 ± 0.28	5.30; 5.49	5.40 ± 0.34	5.30; 5.50	**5.72 ± 0.57**$^{\delta}$	5.52; 5.93	**0.001**	**<0.0001**
Glucose (mg/dL)	85.51 ± 40.41	81.94; 89.10	87.08 ± 8.24	84.71; 89.44	**93.97 ± 20.60***	86.54; 101.40	**0.024**	**0.046**
Insulin (μU/ml)	9.05 ± 4.38	7.54; 10.55	**12.07 ± 5.52***	10.49; 13.65	**20.60 ± 11.8***$^{\delta}$	16.35; 24.85	**<0.0001**	**<0.0001**
HOMA-B	164.22 ± 100.86	129.57; 198.86	210.82 ± 143.58	169.57; 252.05	**282.44 ± 177.43***	218.35; 346.52	**0.004**	**<0.0001**
HOMA-S	64.71 ± 28.99	54.67 ; 79.67	**46.58 ± 19.96***	40.84; 52.31	**30.92 ± 22.6***$^{\delta}$	22.77; 39.07	**<0.0001**	**<0.0001**
HOMA-IR	1.93 ± 0.98	1.59; 2.26	**2.61 ± 1.28***	2.24; 2.97	**4.85 ± 3.08***$^{\delta}$	3.74; 5.96	**<0.0001**	**<0.0001**
Gut microbiota and metabolites								
CAG-Prevotella	0.13 ± 0.19	0.07; 0.2	0.21 ± 0.27	0.13; 0.28	0.14 ± 0.2	0.06; 0.21	0.793	0.920
CAG-Lachnospiraceae	0.20 ± 0.22	0.13; 0.28	0.23 ± 0.22	0.16; 0.29	0.27 ± 0.25	0.18; 0.36	0.781	0.481
CAG-Pathogen	0.10 ± 0.20	0.03; 0.17	0.20 ± 0.26	0.12; 0.27	0.23 ± 0.29	0.12; 0.33	**0.023**	0.060
CAG-Akkermansia	0.25 ± 0.29	0.15; 0.35	0.15 ± 0.23	0.09; 0.22	0.15 ± 0.21	0.07; 0.22	0.120	0.183
CAG-Ruminococcaceae	0.15± 0.16	0.10; 0.21	0.09 ± 0.13	0.05; 0.13	0.08 ± 0.12	0.04; 0.12	**0.047**	0.066
TMA (μM)	1.93 ± 0.62	1.71; 2.14	1.88 ± 0.66	1.69; 2.07	1.77 ± 0.67	1.53; 2.02	0.539	0.616
TMA-O (μM)	3.66 ± 0.53	3.48 3.85	3.61 ± 0.55	3.45; 3.76	3.74 ± 0.57	3.54; 3.95	0.578	0.737
Adipokines and inflammatory markers								
hsCRP (mg/L)	1.50 ± 1.02	1.15; 1.85	**3.44 ± 6.35***	1.62; 5.26	**4.13 ± 3.13***$^{\delta}$	3.00; 5.26	**<0.0001**	**<0.0001**
TNF-α (pg/ml)	13.70 ± 6.55	11.45; 15.95	12.71 ± 7.08	10.68; 14.75	15.85 ± 10.33	12.12; 19.57	0.263	0.093
IL-6 (pg/ml)	6.07 ± 4.56	4.50; 7.64	5.52 ± 4.61	4.20; 6.85	6.87 ± 6.39	4.57; 9.18	0.546	0.299
IL-33 (pg/ml)	130.84 ± 21.73	123.38; 138.31	126.26 ± 16.67	121.47; 131.04	128.56 ± 17.84	122.12; 134.99	0.612	0.932
IL-8 (pg/ml)	29.96 ± 42.25	15.45; 44.47	31.82 ± 40.64	20.15; 43.5	55.51 ± 106.83	16.99; 94.03	0.445	0.183
MCP-1 (pg/ml)	249.29 ± 137.28	202.13; 296.45	257.05 ± 125.55	220.99; 293.11	**302.33 ± 116.24***	260.42; 344.23	**0.039**	**0.010**
IL-1β (pg/ml)	11.43 ± 1.67	10.86; 12.01	11.08 ± 2.24	10.44; 11.72	**12.57 ± 2.62**$^{\delta}$	11.63; 13.51	**0.019**	**0.040**
Visfatin (ng/ml)	3.20 ± 2.84	2.23; 4.18	2.51 ± 2.49	1.80; 3.23	3.04 ± 2.78	2.04; 4.04	0.265	0.349
Resistin (ng/ml)	12.69 ± 4.53	11.13; 14.24	12.48 ± 4.81	11.10; 13.86	13.70 ± 7.17	11.12; 16.28	0.705	0.599
Lipocalin-2 (ng/ml)	29.07 ± 4.76	27.44; 30.70	29.42 ± 4.87	28.02; 30.82	27.24 ± 4.7	25.54; 28.93	0.117	0.073
CXCL5 (ng/ml)	1.32 ± 0.91	1.01; 1.63	1.34 ± 1.18	1.01; 1.68	1.62 ± 0.85	1.31; 1.93	0.178	0.267
Chemerin (ng/ml)	7.33 ± 2.06	6.62; 8.04	**9.05 ± 3.49***	8.04; 10.05	**9.71 ± 3.70***	8.38; 11.04	**0.014**	**0.010**
Vaspin (ng/ml)	2.98 ± 7.35	0.45; 5.50	2.24 ± 7.72	0.03; 4.46	4.55 ± 10.19	0.88; 8.22	0.312	0.246
IL-18 (pg/ml)	251.11 ± 103.61	215.52; 286.70	**307.93 ± 119.19**	273.70; 342.17	**298.73 ± 128.24**	252.5; 344.97	0.065	**0.033**
Leptin (ng/ml)	4.23 ± 4.84	2.56; 5.89	**6.04 ± 5.94***	4.34; 7.75	**11.99 ± 6.68***$^{\delta}$	9.58; 14.4	**<0.0001**	**<0.0001**
Adiponectin (μg/ml)	8.37 ± 3.59	7.14; 9.60	**5.68 ± 3.24***	4.75; 6.62	**4.99 ± 2.04***	4.26; 5.73	**<0.0001**	**<0.0001**
Adiponectin/Leptin	4.91 ± 5.12	3.15; 6.67	**1.96 ± 2.35***	1.29; 2.64	**0.65 ± 0.76***$^{\delta}$	0.38; 0.92	**<0.0001**	**<0.0001**

BMI: body mass index; HDL: high-density lipoprotein cholesterol; VLDL: very low-density lipoprotein cholesterol; LDL: low-density lipoprotein cholesterol; oxLDL: oxidized low-density lipoprotein cholesterol; ApoB: Apolipoprotein B; TMA: Trimethylamine; TMA-O: Trimethylamine N-oxide; HbA1c: glycated hemoglobin; HOMA: homeostasis model assessment; CAG co-abundance groups; hsCRP high sensitivity C reactive protein; TNF-α: Tumor Necrosis Factor alpha; IL: Interleukin; MCP-1: Monocyte Chemoattractant Protein-1; CXCL5: C-X-C motif chemokine ligand 5. Model 1. MLR unadjusted. Model 2 MLR adjusted for potential confounders age range, sex at birth, smoking, and city of origin.

Table S3. Health information, anthropometry, and blood chemistry of subjects grouped based on co-abundant bacterial consortia dominating their gut microbiota. Data presented as the mean ± SD. To compare groups, a multiple linear regression model (MLR) with log-transformed variables was used. The model was adjusted for age range, sex at birth, and city of origin was used. Reported p-values < 0.05 indicate the statistical significance of these comparisons. Post hoc analyses employing Dunn's test were conducted for individual contrasts. Differences were considered statistically significant if the adjusted p-value was less than 0.05. (*) indicates a statistically significant difference between the gut microbiotas dominated by CAG-Ruminococcaceae and CAG-Pathogen. ($^{\delta}$) indicates the contrast CAG-Akkermansia and CAG-Pathogen. ¥ indicates the contrast of CAG-Pathogen and CAG-Lachnospiraceae.

	**CAG–Ruminococcaceae**		**CAG–Akkermansia**		**CAG–Prevotella**		**CAG–Lachnospiraceae**		**CAG–Pathogen**		**p–value**	
	**Mean ± SD**	**CI 95%**	**Mean ± SD**	**CI 95%**	**Mean ± SD**	**CI 95%**	**Mean ± SD**	**CI 95%**	**Mean**	**CI 95%**		
**N**	20		23		22		27		24		Model 1	Model 2
Demographics												
Age (years)	42.3		43.4		36.3		39.1		42.3			
Female	13		16		9		10		8			
male	7		7		13		17		16			
Anthropometry												
BMI (kg/m^2)	26.04 ± 4.08	24.12; 27.95	26.20 ± 4.44	24.28; 28.12	29.01 ± 6.08	26.31; 31.71	28.67 ± 4.38	26.94; 30.40	28.57 ± 3.78	26.98; 30.17	0.059	0.046
Weight (kg)	68.65 ± 14.64	61.79; 75.50	67.28 ± 14.26	61.11; 73.45	78.72 ± 17.18	71.10; 86.34	78.70 ± 14.35	73.02; 84.37	79.20 ± 13.59	73.47; 84.94	**0.003**	0.053
Waist circumference (cm)	89.68 ± 11.20	84.43; 94.92	87.97 ± 13.86	81.97; 93.96	94.43 ± 13.70	88.35; 100.5	97.14 ± 11.41	92.63; 101.66	97.97 ± 12.92	92.52; 103.43	**0.018**	0.070
Body Fat (%)	38.28 ± 5.61	35.65; 40.90	37.07 ± 4.27	35.22; 38.92	37.58 ± 5.94	34.95; 40.21	37.44 ± 5.65	35.21; 39.67	36.25 ± 5.19	34.06; 38.44	0.853	0.267
Blood pressure												
Systolic BP (mm Hg)	116.9 ± 14.49	110.07; 123.63	119.60 ± 15.60	112.82; 126.31	122.40 ± 18.47	114.17; 130.55	132.37 ± 20.12	124.41; 140.33	**133.92 ± 20.88***	125.10; 142.74	**0.003**	**0.014**
Diastolic BP (mm Hg)	73.95 ± 9.89	69.32; 78.58	77.09 ± 11.47	72.13; 82.04	75.09 ± 11.37	70.05; 80.13	84.52 ± 12.20	79.69; 89.35	**85.38 ± 13.52***	79.67; 91.08	**<0.0001**	**0.002**
Blood lipids												
HDL (mg/dL)	49.15 ± 11.75	43.65; 54.65	47.35 ± 10.39	42.85; 51.84	43.50 ± 8.82	39.59; 47.41	41.15 ± 8.47	37.80; 44.50	42.33 ± 13.81	36.50; 48.16	0.053	0.711
VLDL (mg/dL)	23.79 ± 10.20	19.02; 28.56	30.93 ± 32.85	16.73; 45.13	24.25 ± 10.42	19.63; 28.86	32.16 ± 19.26	24.54; 39.78	35.99 ± 25.30	25.30; 46.67	0.195	0.342
LDL (mg/dL)	119.40 ± 34.85	103.09; 135.71	113.60 ± 34.38	98.70; 128.43	112.80 ± 27.16	100.73; 124.82	116.73 ± 28.20	105.34; 128.12	124.08 ± 41.51	106.56; 141.61	0.836	0.606
Total cholesterol (mg/dL)	189.60 ± 38.57	171.55; 207.65	189.80 ± 43.44	171; 208.57	177.40 ± 31.30	163.49; 191.24	187.11 ± 35.10	173.22; 201.00	199.46 ± 45.16	180.39; 218.53	0.510	0.639
Triglycerides (mg/dL)	118.90 ± 50.82	95.07; 142.63	158.1 ± 163.6	87.37; 228.89	121.1 ± 51.76	98.14; 144.04	160.85 ± 96.41	122.71; 198.99	180.04 ± 126.7	126.54; 233.54	0.206	0.317
oxLDL (U/L)	160.10 ± 102.50	112.11; 208.07	152.3 ± 65.79	123.89; 180.79	166.7 ± 88.56	125.22; 208.11	170.62 ± 118.10	121.86; 219.39	190.50 ± 135.00	132.11; 248.89	0.857	0.795
ApoB (mg/dL)	94.86 ± 27.34	82.06; 107.65	90.23 ± 24.82	79.49; 100.96	113.50 ± 132.80	54.59; 172.35	102.10 ± 29.37	90.49; 113.72	99.20 ± 23.40	89.32; 109.08	0.733	0.682
Blood sugar control												
HbA1c (%)	5.60 ± 0.42	5.41; 5.80	5.50 ± 0.55	5.26; 5.74	5.48 ± 0.31	5.34; 5.61	5.38 ± 0.38	5.23; 5.53	5.51 ± 0.44	5.32; 5.70	0.487	0.690
Glucose (mg/dL)	90.45 ± 11.70	84.98; 95.92	88.56 ± 22.99	78.62; 98.51	86.59 ± 8.72	82.72 - 90.46	86.85 ± 7.93	83.72-89.99	90.46 ± 12.63	85.11 -90.46	0.694	0.560
Insulin (µU/ml)	12.69 ± 5.58	8.67; 16.71	11.95 ± 6.73	9.05;14.87	13.02 ± 7.87	9.53-16.52	15.28 ± 12.20	10.46-20.11	14.14 ± 6.89	11.23 -17.05	0.730	0.460
HOMA-B	172.14 ± 95.92	127.25; 217.04	212.56 ± 129.15	156.72; 268.41	202.0 ± 99.59	158.04; 246.36	258.79 ± 211.38	175.17; 342.41	222.83 ± 154.05	157.78; 287.88	0.728	0.161
HOMA-S	50.82 ± 29.83	36.86; 64.78	53.21 ± 31.10	39.76; 66.65	49.73 ± 28.69	37.01; 62.45	46.02 ± 25.44	35.96; 56.08	39.99 ± 18.61	32.14; 47.85	0.734	0.603
HOMA-IR	3.01 ± 2.73	1.73; 4.28	2.68 ± 1.73	1.94; 3.43	2.90 ± 2.08	1.97; 3.82	3.29 ± 2.75	2.20; 4.38	3.17 ± 1.66	2.47; 3.87	0.735	0.605
Gut microbiota and metabolites												
CAG- Prevotella	0.09 ± 0.06	0.06; 0.11	0.02 ± 0.02	0.01; 0.03	0.60 ± 0.16	0.53; 0.67	0.08 ± 0.07	0.05; 0.11	0.07 ± 0.10	0.03; 0.11	**<0.0001**	**<0.0001**
CAG-Lachnospiraceae	0.15 ± 0.07	0.12; 0.19	0.11 ± 0.06	0.08; 0.14	0.10 ± 0.08	0.07; 0.14	0.61 ± 0.15	0.55; 0.67	**0.11 ± 0.09*¥**	0.08; 0.15	**<0.0001**	**<0.0001**
CAG-Pathogen	0.03 ± 0.04	0.02; 0.05	0.05 ± 0.07	0.02; 0.08	0.06 ± 0.08	0.02; 0.09	0.08 ± 0.07	0.05; 0.10	**0.64 ± 0.17***	0.57; 0.71	**<0.0001**	**<0.0001**
CAG-Akkermansia	0.10 ± 0.08	0.06; 0.14	0.64 ± 0.13	0.58; 0.70	0.06 ± 0.06	0.03; 0.09	0.07 ± 0.08	0.04; 0.10	**0.05 ± 0.06$^{\delta}$**	0.03; 0.08	**<0.0001**	**<0.0001**
CAG-Ruminococcaceae	0.37 ± 0.05	0.35; 0.40	0.07 ± 0.08	0.03; 0.11	0.05 ± 0.05	0.03; 0.07	0.04 ± 0.07	0.02; 0.07	**0.04 ± 0.04***	0.02; 0.05	**<0.0001**	**<0.0001**
TMA (µM)	1.83 ± 0.70	1.50; 2.16	1.92 ± 0.62	1.65; 2.18	1.72 ± 0.50	1.50; 1.94	2.01 ± 0.7	1.73; 2.28	1.81 ± 0.70	1.51; 2.11	0.671	0.818
TMA-O (µM)	3.60 ± 0.46	3.39; 3.82	3.66 ± 0.60	3.40; 3.92	3.39 ± 0.49	3.17; 3.61	3.83 ± 0.52	3.62; 4.03	3.78 ± 0.58	3.53; 4.02	0.067	0.178
Adipokines and inflammatory markers												
hsCRP (mg/L)	1.77 ± 0.93	1.34; 2.21	2.26 ± 1.41	1.65; 2.87	2.79 ± 2.43	1.71; 3.87	4.47 ± 8.20	1.23; 7.71	3.50 ± 3.96	1.82; 5.17	0.257	0.296
TNF–α¥ (pg/ml)	16.24 ± 5.27	13.77; 18.71	12.73 ± 7.16	9.63; 15.83	10.14 ± 3.58	8.55; 11.73	17.43 ± 10.18 $^{\delta}$	13.41; 21.46	12.47 ± 9.10	8.62; 16.31	**0.001**	0.963
IL-6 (pg/ml)	7.70 ± 4.04	5.81; 9.59	5.40 ± 4.64	3.39; 7.40	3.86 ± 2.44	2.78; 4.94	8.12 ± 6.45	5.57; 10.68	5.06 ± 5.62	2.68; 7.43	**0.001**	0.958
IL–33 (pg/ml)	137.60 ± 14.66	130.71; 144.43	124.7 ± 15.46	118.02; 131.39	120.8 ± 14.37	114.41; 127.15	136.24 ± 24.82	126.42; 146.06	121.85 ± 13.25	116.26; 127.44	**0.002**	0.723
IL-8 (pg/ml)	31.54 ± 37.47	14.00; 49.07	32.22 ± 51.63	9.9; 54.55	39.77 ± 36.94	23.40; 56.15	45.81 ± 98.83	6.71; 84.91	37.46 ± 76.99	4.95; 69.97	0.790	0.571
MCP-1 (pg/ml)	247.20 ± 125.10	188.67; 305.78	271.30 ± 144.70	208.76; 333.9	268.80 ± 90.5	228.64; 308.89	283.48 ± 130.30	231.94; 335.01	260.15 ± 145.1	198.9; 321.39	0.748	0.676
IL1β (pg/ml)	12.60 ± 1.68	11.81; 13.39	11.31 ± 2.03	10.43; 12.19	11.02 ± 1.82	10.21; 11.82	12.38 ± 2.73	11.31; 13.46	10.68 ± 2.26	9.73; 11.63	0.010	0.799

Visfatin (ng/ml)	3.06 ± 2.26	2.00; 4.12	2.15 ± 2.49	1.07; 3.22	2.21 ± 1.95	1.35; 3.08	3.79 ± 2.99	2.60; 4.97	2.95 ± 3.18	1.61; 4.30	0.814	0.924
Resistin (ng/ml)	10.82 ± 2.17	9.81; 11.83	12.32 ± 3.65	10.74; 13.90	15.18 ± 8.9	11.23; 19.12	12.94 ± 4.12	11.31; 14.57	12.95 ± 5.65	10.56; 15.33	0.502	0.264
Lipocalin-2 (ng/ml)	28.94 ± 5.36	26.43; 31.45	28.43 ± 4.54	26.47; 30.40	29.15 ± 4.75	27.04; 31.25	29.34 ± 4.49	27.56; 31.11	27.69 ± 5.31	25.45; 29.93	0.701	0.337
CXCL5 (ng/ml)	1.47 ± 0.91	1.05; 1.90	1.48 ± 0.91	1.08; 1.87	1.31 ± 0.75	0.98; 1.64	1.38 ± 0.81	1.07; 1.70	1.43 ± 1.56	0.77; 2.09	0.944	0.988
Chemerin (ng/ml)	7.16 ± 2.15	6.15; 8.17	8.58 ± 2.39	7.55; 9.61	9.15 ± 3.04	7.80; 10.50	9.06 ± 3.90	7.52; 10.61	9.34 ± 4.12	7.6; 11.08	0.260	0.776
Vaspin (ng/ml)	3.52 ± 8.42	–0.42; 7.46	1.78 ± 4.84	–0.31; 3.87	3.50 ± 9.43	–0.68; 7.69	4.77 ± 11.86	0.08; 9.46	1.77 ± 4.50	–0.13; 3.67	0.823	0.634
IL–18 (pg/ml)	219.30 ± 61.91	190.33; 248.28	252.20 ± 95.91	210.7; 293.65	278.70 ± 124.8	223.38; 334.07	**347.96 ± 137.40** $^{\delta}$	293.62; 402.30	**321.84 ± 111.20***	274.91; 368.78	**0.002**	**0.025**
Leptin (ng/ml)	6.76 ± 4.73	4.54; 8.97	7.63 ± 6.62	4.77; 10.49	7.03 ± 6.88	3.98; 10.08	7.15 ± 7.25	4.28; 10.02	7.05 ± 7.30	3.97; 10.13	0.468	0.905
Adiponectin (µg/ml)	7.39 ± 3.15	5.91; 8.86	7.65 ± 4.78	5.58; 9.72	5.68 ± 1.60	4.97; 6.39	5.51 ± 2.60	4.48; 6.54	5.57 ± 3.43	4.12; 7.02	0.053	0.415
Adiponectin/leptin	2.07 ± 1.98	1.14; 2.99	1.41 1.10	0.93; 1.88	3.90 ± 5.29	1.55; 3.24	2.84 4.63	1.01; 4.67	2.20 ± 2.79	1.02; 3.37	0.816	0.693

BMI: body mass index; HDL: high-density lipoprotein cholesterol; VLDL: very low-density lipoprotein cholesterol; LDL: low-density lipoprotein cholesterol; oxLDL: oxidized low-density lipoprotein cholesterol; ApoB: Apolipoprotein B; TMA: Trimethylamine; TMA-O: Trimethylamine N-oxide; HbA1c: glycated hemoglobin; HOMA: homeostasis model assessment; CAG co-abundance groups; hsCRP high sensitivity C reactive protein; TNF-α: Tumor Necrosis Factor alpha; IL: Interleukin; MCP-1: Monocyte Chemoattractant Protein-1; CXCL5: C-X-C motif chemokine ligand 5. Model 1. MLR unadjusted. Model 2 MLR adjusted for potential confounders age range, sex at birth, smoking, and city of origin.

Table S4. Health information, anthropometry, blood chemistry, and gut microbiota status of subjects categorized according to their cardiometabolic health status (healthy vs. abnormal). The data are presented as the mean ± SD and 95% CIs. To compare groups, a multiple linear regression model (MLR) with log-transformed variables was used. The model was adjusted for age range, sex at birth, and city of origin. Reported p-values <0.05 indicate the statistical significance of these comparisons.

	**Cardiometabolically healthy**		**Cardiometabolically abnormal**		**p-value**	
	**Mean ± SD**	**CI 95%**	**Mean ± SD**	**CI 95%**		
**N**	44		72		Model 1	Model 2
Demographics						
Age (Years)	39.4		41.4			
Female	22		34			
male	22		38			
Anthropometry						
BMI (kg/m^2)	25.13 ± 3.46	24.07; 26.18	**29.42 ± 4.62**	28.33; 30.51	**<0.0001**	**<0.0001**
Weight (kg)	67.2 ± 12.99	63.25; 71.15	**79.46 ± 15.13**	75.91; 83.02	**<0.0001**	**<0.0001**
Waist Circumference (cm)	85.98 ± 10.11	82.91; 89.05	**98.4 ± 12.46**	95.48; 101.33	**<0.0001**	**<0.0001**
Body Fat (%)	35.59 ± 4.92	34.1; 37.09	**38.33 ± 5.3**	37.08; 39.57	**0.009**	**<0.0001**
Blood pressure						
Systolic (mm Hg)	116.59 ± 14.8	112.09; 121.09	**131.07 ± 19.65**	126.45; 135.69	**<0.0001**	**<0.0001**
Diastolic (mm Hg)	73.8 ± 10.31	70.66; 76.93	**83.17 ± 12.59**	80.21; 86.13	**<0.0001**	**<0.0001**
Blood lipids						
HDL (mg/dL)	50.68 ± 8.17	48.2; 53.17	**40.64 ± 10.82**	38.1; 43.18	**<0.0001**	**<0.0001**
VLDL (mg/dL)	20.24 ± 8.37	17.69; 22.78	**35.59 ± 25.29**	29.64; 41.53	**<0.0001**	**<0.0001**
LDL (mg/dL)	119.18 ± 33.56	108.98; 129.39	116.9 ± 33.55	109.02; 124.79	0.686	0.693
TC (mg/dL)	187.43 ± 39.34	175.47; 199.39	189.6 ± 39.06	180.42; 198.78	0.743	0.948
TG (mg/dL)	101.2 ± 41.64	88.54; 113.87	**179.01 ± 126.13**	149.38; 208.65	**<0.0001**	**<0.0001**
ox-LDL (U/L)	156.9 ± 68.44	136.09; 177.71	176.83 ± 119.46	148.76; 204.9	0.627	0.323
ApoB (mg/dL)	90.96 ± 26.72	82.84; 99.09	105.61 ± 75.38	87.89; 123.32	0.106	0.082
Blood sugar control						
Glucose (mg/dL)	84.07 ± 6.02	82.24; 85.90	**91.22 ± 16.15**	87.43; 95.02	0.168	**0.007**
HbA1c (%)	5.41 ± 0.31	5.32; 5.51	**5.53 ± 0.48**	5.42; 5.64	**0.004**	**0.019**
Insulin (µU/ml)	8.41 ± 2.64	7.61; 9.22	**16.63 ± 9.71**	14.34; 18.91	**<0.0001**	**<0.0001**
HOMA-B	156.78 ± 77.30	133.28; 180.28	**253.01 ± 169.53**	213.17; 292.85	**0.002**	**<0.0001**
HOMA-S	64.16 ± 23.68	56.96; 71.36	**37.69 ± 23.58**	32.14; 43.23	**<0.0001**	**<0.0001**
HOMA-IR	1.75 ± 0.57	1.58; 1.92	**3.80 ± 2.48**	3.22; 4.38	**<0.0001**	**<0.0001**
Gut microbiota and metabolites						
CAG-Prevotella	0.21 ± 0.25	0.13; 0.29	0.14 ± 0.21	0.09; 0.19	0.426	0.373
CAG-Lachnospiraceae	0.2 ± 0.22	0.13; 0.27	0.25 ± 0.23	0.2; 0.31	0.197	0.225
CAG-Pathogen	0.09 ± 0.15	0.04; 0.13	**0.23 ± 0.29**	0.16; 0.3	**0.027**	0.130
CAG-Akkermansia	0.21 ± 0.26	0.13; 0.29	0.17 ± 0.23	0.11; 0.22	0.317	0.676
CAG-Ruminococcaceae	0.14 ± 0.16	0.09; 0.19	0.08 ± 0.12	0.06; 0.11	0.130	0.192
TMA (µM)	1.83 ± 0.64	1.64; 2.03	1.88 ± 0.65	1.72; 2.04	0.663	0.490
TMA-O (µM)	3.65 ± 0.58	3.47; 3.72	3.67 ± 0.53	3.54; 3.79	0.805	0.954
Adipokines and inflammatory markers						
hsCRP (mg/L)	1.58 ± 1.04	1.26; 1.89	**3.94 ± 5.57**	2.64; 5.25	**<0.0001**	**<0.0001**
TNF-α (pg/ml)	13.89 ± 6.26	11.98; 15.79	13.87 ± 8.94	11.77; 15.97	0.560	0.769
IL-6 (pg/ml)	6.08 ± 4.36	4.75; 7.4	6.05 ± 5.58	4.74; 7.36	0.668	0.815
IL-33 (pg/ml)	130.95 ± 19.02	125.17; 136.73	126.64 ± 18.24	122.35; 130.93	0.215	0.183
IL-8 (pg/ml)	32.12 ± 40	19.96; 44.29	41.26 ± 78.42	22.83; 59.69	0.762	0.187
MCP-1 (pg/ml)	252.79 ± 135.19	211.69; 293.89	276.00 ± 122.84	247.14; 304.87	0.177	0.129
IL-1β (pg/ml)	11.54 ± 1.92	10.96; 12.13	11.63 ± 2.47	11.05; 12.21	0.971	0.475
Visfatin (ng/ml)	2.48 ± 2.2	1.81; 3.15	3.10 ± 2.92	2.42; 3.79	0.428	0.673
Resistin (ng/ml)	12.78 ± 5.16	11.21; 14.35	12.94 ± 5.67	11.6; 14.27	0.975	0.586
Lipocalin-2 (ng/ml)	29.01 ± 5.25	27.42; 30.61	28.53 ± 4.6	27.45; 29.61	0.660	0.665
CXCL5 (ng/ml)	1.36 ± 0.88	1.10; 1.63	1.44 ± 1.1	1.18; 1.7	0.760	0.865
Chemerin (ng/ml)	7.8 ± 2.55	7.03; 8.58	**9.27 ± 3.6**	8.42; 10.11	**0.024**	**0.013**
Vaspin (ng/ml)	2.94 ± 7.81	0.57; 5.31	3.20 ± 8.72	1.15; 5.25	0.610	0.953
IL-18 (pg/ml)	260.51 ± 96.68	231.12; 289.9	305.21 ± 128.42	275.03; 335.38	0.096	0.107
Leptin (ng/ml)	4.65 ± 3.83	3.49; 5.82	**8.65 ± 7.42**	6.9; 10.39	**<0.0001**	**<0.0001**
Adiponectin (µg/ml)	7.47 ± 3.32	6.46; 8.48	**5.59 ± 3.20**	4.84; 6.34	**<0.0001**	**<0.0001**
Adiponectin/Leptin	4.32 ± 5.03	2.79;5.85	**1.37 ± 1.57**	1.00; 1.74	**<0.0001**	**<0.0001**

BMI: body mass index; HDL: high-density lipoprotein cholesterol; VLDL: very low-density lipoprotein cholesterol; LDL: low-density lipoprotein cholesterol; oxLDL: oxidized low-density lipoprotein cholesterol; ApoB: Apolipoprotein B; TMA: Trimethylamine; TMA-O: Trimethylamine N-oxide; HbA1c: glycated hemoglobin; HOMA: homeostasis model assessment; CAG co-abundance groups; hsCRP high sensitivity C reactive protein; TNF-α: Tumor Necrosis Factor alpha; IL: Interleukin; MCP-1: Monocyte Chemoattractant Protein-1; CXCL5: C-X-C motif chemokine ligand 5. Model 1. MLR unadjusted. Model 2 MLR adjusted for potential confounders age range, sex at birth, smoking, and city of origin.

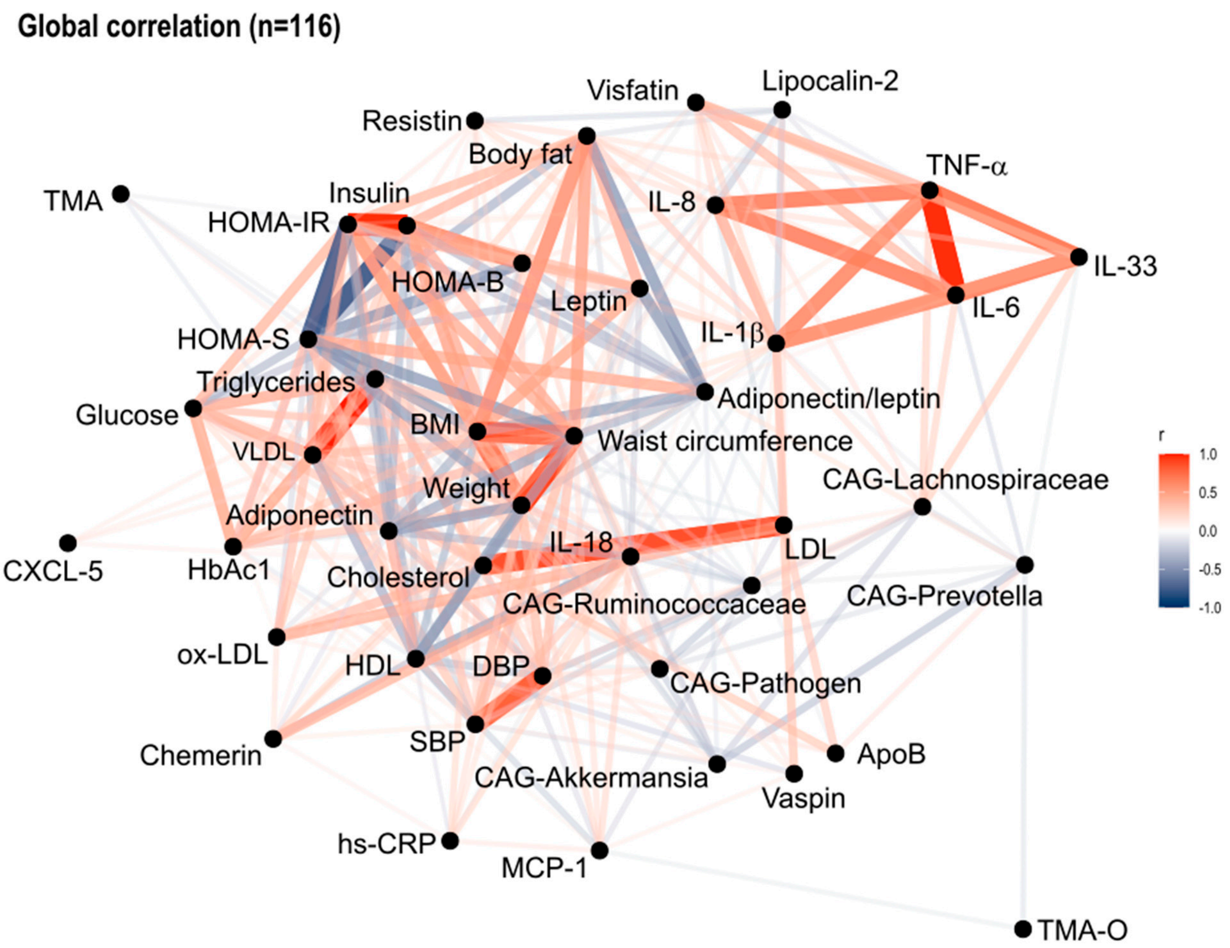

Figure S1. The correlation network of all variables (CAGs, blood chemistry, and adipokines) evaluated in 116 subjects**;** red lines stand for positive correlations, and blue lines for negative correlations ($r^2 > 0.2$). BMI: body mass index, TC: total cholesterol, TG: triglycerides, HDL: high-density lipoprotein cholesterol, LDL: low-density lipoprotein cholesterol, VLDL: very low-density lipoprotein cholesterol, HOMA-IR: homeostatic model assessment–insulin resistance, hs-CRP: high sensitive C-reactive protein. BP: blood pressure (systolic and diastolic).
